# The interrelationship between women’s help-seeking experiences for vaginismus and their sense of self: a qualitative study and abductive analysis

**DOI:** 10.1080/21642850.2024.2396134

**Published:** 2024-08-29

**Authors:** Rashmi Pithavadian, Tinashe Dune, Jane Chalmers, Vijayasarathi Ramanathan

**Affiliations:** a School of Health Sciences, Western Sydney University, Penrith, Australia; b Translational Health Research Institute, Western Sydney University, Penrith, Australia; c Allied Health and Human Performance, University of South Australia, Adelaide, Australia; d Faculty of Medicine, University of Sydney, Camperdown, Australia

**Keywords:** Vaginismus, help-seeking, sense of self, qualitative study, women's health‌

## Abstract

**Objective:**

There is a lack of research on women’s holistic experiences of vaginismus, also called sexual pain-penetration disorder, from their perspective. To address this gap, an abductive qualitative study aimed to examine women’s help-seeking experiences for vaginismus, and its impact on their sense of self.

**Methods:**

This study was informed by a feminist approach to the theory of self focused on participants’ negotiation of dis/empowerment when help-seeking for vaginismus. Twenty-one participants aged 19–37 years (mean 27.6 years) and diagnosed with vaginismus in Australia participated in semi-structured interviews, which were thematically analysed.

**Results:**

Three themes were developed: *Interconnected constructions of womanhood and help-seeking*, *Elicit agency to move forward*, *Resilience to surmount challenges* with subthemes. Participant’s overall help-seeking experiences, within and outside the healthcare system, shaped their sense of self in ways that drove their approach/es to future help-seeking behaviours.

**Conclusions:**

Positive help-seeking experiences for vaginismus strengthened participants’ sense of self to persevere with treatment even when it was difficult. Conversely, negative help-seeking experiences led to participants’ weakened sense of self which was often caused by a gap between their ideal and perceived self. This led to negative feelings and responses of demotivation or halting subsequent help-seeking. Recommendations are provided to improve health professional practice to support women help-seeking for vaginismus, and to help close the gap between their ideal and perceived selves. Such insight can help to empower women’s sense of self and motivate them to persevere with help-seeking to experience improvement for their vaginismus and quality of life.

## Introduction

### Impact and prevalence of vaginismus

Vaginismus is a sexual pain disorder that can make any type of vaginal penetration to be painful, difficult, or impossible (Ramanathan et al., 2022). It can cause an inability for women to consummate their relationships, and make it difficult to start a family (McEvoy et al., 2021). Women’s vaginismus also has emotional, mental, and physical consequences on their male partners, which increases their risk of developing male sexual dysfunction (Eserdag et al., 2021). Therefore, women with vaginismus face a high likelihood of relationship stress and breakdowns (McEvoy et al., 2021). Vaginismus also impedes people’s sexuality in terms of sexual expression and exploration of penetrative activities (Alizadeh & Farnam, 2021). The condition can reduce women’s self-esteem and self-contentment, and increase their social withdrawal, anxiety, depression, and suicidal ideation (Velayati et al., 2021; Sorensen et al., 2018; Chew et al., 2021). All these factors can have a domino effect and significantly reduce people’s quality of life (Alizadeh & Farnam, 2021).

Vaginismus has an estimated prevalence ranging from 1% to 7% of women in the general population (Yilmaz et al., 2020). However, prevalence among women is reported higher in countries such as Turkey, Ghana, and Iran (McEvoy et al., 2021). Prevalence rates are difficult to determine. This is due to stigma towards discussing sexual health, those who do not seek help not being counted in reporting of prevalence, and low social awareness of vaginismus (McEvoy et al., 2021; Velayati et al., 2021; Laskowska & Gronowski, 2022). While vaginismus is most common in those who identify as women, it can affect anyone with a vagina regardless of their gender identity.

### Terminology of vaginismus

Vaginismus is defined as ‘spasm of the pelvic floor muscles that surround the vagina, causing occlusion of the vaginal opening. Penile entry is either impossible or painful’ in the International Classification of Diseases 10 (ICD-10) (World Health Organization, 1994). In the ICD-11 that came into effect in 2022, vaginismus has been revised into sexual pain-penetration disorder (SPPD) (World Health Organization, 2019; Reed et al., 2016). SPPD is defined by one or more of the following symptoms: persistent difficulty of vaginal penetration, recurrent pain during attempts at vaginal penetration, and fear or anxiety of pain associated with vaginal penetration (World Health Organization, 2019).

There is a dichotomy between the ICD’s and Diagnostic and Statistical Manual’s (DSM) approach to vaginismus in relation to dyspareunia, which refers to painful sex (World Health Organization, 1994; World Health Organization, 2019; Reed et al., 2016; American Psychiatric Association, 2022). Vaginismus was combined with dyspareunia and renamed to genito-pelvic pain/penetration disorder (GPPPD) in the DSM-V (Reed et al., 2016; American Psychiatric Association, 2022). Whereas, the ICD-10 has vaginismus as a separate diagnostic category to dyspareunia. Moreover, unlike GPPPD’s merging, even the new SPPD in the ICD-11 lists the diagnostic category of dyspareunia, when caused by physical issues, as an exclusion (World Health Organization, 2019; Reed et al., 2016). This study uses the ICD-10 terminology of vaginismus because its diagnostic criteria was in usage at the time of participant recruitment before 2022. Moreover, many countries around the world still use versions of the ICD-10 (Otero Varela et al., 2021). The use of vaginismus is also appropriate because the term continues to be used in clinical settings (Ramanathan et al., 2022; Pithavadian, Chalmers et al., 2023a), and recent research (Chalmers, 2024; Raveendran & Rajini, 2024; Banaei et al., 2023).

The location of pain, psychological or physiological symptoms, and treatment varies between vaginismus and other dyspareunic conditions such as vulvodynia, endometriosis, and adenomyosis (Vieira-Baptista et al., 2018). Therefore, it is not always helpful to merge these conditions. This study’s use of the ICD-10 terminology allows for exploration of two facets. Firstly, women’s help-seeking experiences for vaginismus can be examined as a singular pelvic health issue. Secondly, it allows nuanced consideration of how women with vaginismus can have their well-being and health management further affected by other dyspareunic co- or multi-morbidities, such as endometriosis or vulvodynia.

### Help-seeking for vaginismus and androcentrism in health systems

Help-seeking refers to searching for help to gain information, diagnosis, treatment, relief, or support for a presenting health issue in healthcare contexts (Rickwood & Thomas, 2012). Formal help-seeking involves assistance from trained professionals (Rickwood & Thomas, 2012; Stunden et al., 2020). Informal help-seeking refers to seeking assistance from people who have personal and non-professional relations such as friends and family (Rickwood & Thomas, 2012; Stunden et al., 2020). Informal help-seeking exists outside the healthcare system, and more recently includes using the internet to seek health information (Macey et al., 2015).

Women face various challenges when trying to seek and receive help for their vaginismus. The low social and medical awareness of vaginismus among health professionals is a barrier for women to receive help for the condition (Pacik et al., 2019). Some health professionals lack understanding to make appropriate diagnosis and referrals. Women have to often consult various sources, which takes years to gain an appropriate diagnosis of vaginismus (Pithavadian, Chalmers et al., 2023a).

The androcentric nature of the healthcare system impedes help-seeking (Shallcross et al., 2019; Merone et al., 2022). Women’s concerns of painful sex are often dismissed or minimised by health professionals, who sometimes even give generic verbal advice to ‘relax’ to relieve symptoms (Hobbs et al., 2019). This often leads to women receiving misdiagnosis, incorrect treatment, or misinformation regarding their vaginismus (Kingsberg et al., 2019; Pacik & Geletta, 2017). Ethnically and racially diverse women face intersectional and compounding barriers of gendered and racial stereotypes when they seek help for sexual pain (Thorpe et al., 2022). For example, Black women are perceived as superhuman, strong, and less susceptible to pain (Thorpe et al., 2022). Therefore, ethnically and racially diverse women are taken less seriously, misjudged, and misunderstood by health professionals (Thorpe et al., 2022). Negative interactions with health professionals and the healthcare system has led to women taking breaks from help-seeking, or halting help-seeking altogether and struggling with their symptoms and lowered quality of life (Pithavadian, Chalmers et al., 2023a).

Women also consult family, friends, and increasingly the internet, as common sources to seek help for vaginismus (Hobbs et al., 2019). However, the veracity and impact of information received from such informal help-seeking sources is unclear. It should be paramount for women to have positive experiences to gain information, diagnosis, treatment, and support, which will improve their help-seeking outcomes, relationships, sexuality, mental health and overall quality of life (Pithavadian, Chalmers et al., 2023a).

### Help-seeking for vaginismus and sense of self

Women’s help-seeking experiences from formal and informal sources inform their construction of self in ways that have implications for their health and well-being (Ferrè et al., 2014). This is not to say help-seeking is the only factor that influences sense of self, but it is a significant factor worth investigating to improve women’s outcomes for vaginismus. Sense of self is how people perceive themselves as an individual (Chrisler & Johnston-Robledo, 2018). Within this study, it is understood that one’s sense of self is socially constructed. This is because people construct meaning of the world and themselves in relation to societal expectations and social interactions rather than simply in isolation within themselves (Pithavadian et al., 2023b).

Social interactions with health professionals, health systems, and informal sources when help-seeking for vaginismus can have varied outcomes for symptom management and quality of life (Thorpe et al., 2022; Leeds-Hurwitz, 2009). From a social constructivist perspective, women’s social interactions when help-seeking and its varied outcomes inform their constructions of sense of self (Leeds-Hurwitz, 2009). The examination of the association between help-seeking for vaginismus and sense of self greatly expands on some past studies simply analysing the ontology of women’s symptoms of pain and penetration on their sense of self (Velayati et al., 2021; Ward & Ogden, 1994; Stelko, 2015).

### Current literature

There is a lack of research on women’s holistic experiences help-seeking for their vaginismus (Pithavadian, Chalmers et al., 2023a). In line with understandings of holistic health, this refers to reviewing the mental, emotional, social, and spiritual components of health when help-seeking for vaginismus (Zamanzadeh et al., 2015). The lack of such literature on vaginismus and SPPD is due to the fact that current research on the topic tends to be clinically focused on the affected genitalia and aetiology of the condition (Pithavadian, Chalmers et al., 2023a). A recent review focused on help-seeking for vaginismus found that it continues to be a problematic process around the world (Pithavadian, Chalmers et al., 2023a). Out of the 22 included studies, none explicitly examined the impact of people’s help-seeking for vaginismus on their sense of self which was identified as an epistemological gap (Pithavadian, Chalmers et al., 2023a). The review concluded that future research should examine and identify ways to improve women’s help-seeking for vaginismus, its impact on their sense of self, and treatment and healthcare outcomes (Pithavadian, Chalmers et al., 2023a).

Therefore, first addressing the research gap to understand how help-seeking for vaginismus holistically impacts women’s sense of self is paramount. It can provide valuable insight on people’s treatment adherence and factors that drive them to preserve or discontinue future help-seeking. Therefore, this study sought to contribute to the research gap by answering the following research question: What impact does women’s help-seeking for their vaginismus have on their sense of self?

### Theory

A feminist approach to the theory of self informed the development of this study and analysis of findings. Women’s experiences of vaginismus are often dismissed and overlooked (McEvoy et al., 2021; Pithavadian, Chalmers et al., 2023a). The exploration of power is a key tenet of feminist analysis of marginalised groups (Allen, 2018). Therefore, the feminist approach of this study focused on women’s experiences of dis/empowerment when help-seeking for vaginismus and its impact on their sense of self and future health approaches and behaviours (Ardovini-Brooker, 2017; Kiguwa, 2019).

Theory of self was chosen as it did not restrict the study’s analysis to one aspect of self such as self-esteem, self-awareness, or self-worth (Gallagher, 2013). Moreover, theory of self facilitated a more holistic examination of the multifaceted and dynamic nature of self (Gallagher, 2013). Rather than directing participants to focus on a specific aspect of self determined by the researchers, using the broader theory of self allowed more flexibility for the study to consider the multiple aspects of self that participants raised as relevant. This aligned with a feminist approach to theory of self to centre participants’ perspectives to drive conversation on self.

A constructivist philosophy of science underpins the study’s feminist approach to theory of self (Pithavadian et al., 2023b; Gallagher, 2013; Radtke, 2017). Following such constructivism involves perceiving no objective truth (Radtke, 2017). Rather, this approach values women’s constructions of the relationship between their help-seeking experiences and sense of self as providing insightful knowledge to understand and improve future healthcare approaches (Pithavadian, Chalmers et al., 2023a). For clarity, ‘sense of self,’ ‘self’, and ‘construction/s of self’ will be interchanged where appropriate.

## Methods

### Study design

The findings presented in this paper are derived from a larger qualitative study (Pithavadian, 2021; Pithavadian et al., 2024). An abductive qualitative research design, through semi-structured interviews, was used to produce rich data focused on the meanings that participants constructed (Sutton & Austin, 2015). The standards for reporting qualitative research have been followed (O’Brien et al., 2014). Abductive reasoning diverges from deductive approaches that apply analytical frameworks *a priori* and inductive reasoning that seeks to allow theory to emerge from the data (Timmermans & Tavory, 2012). Therefore, this study uses abductive reasoning because it considers multiple theorisations to investigate ‘surprising research evidence’ of either missing or anomalous findings to produce original theoretical contributions (Timmermans & Tavory, 2012, p. 167).

The surprising research evidence that this study investigates with abductive reasoning is the missing data, or research gap, on the association and implications between help-seeking for vaginismus and sense of self (Pithavadian, Chalmers et al., 2023a). A feminist approach to theory of self were the multiple theorisations used, and the other theories considered are discussed as part of data analysis. This study follows Timmermans and Tavory’s three formal methodological steps for abduction by: revisiting the phenomena, defamiliarisation, and alternate casing (Timmermans & Tavory, 2012).

### Ethical statement

The study received ethics approval by the Western Sydney University Human Research and Ethics Committee (Approval Number: H13618). All participants gave informed consent to participate in the study.

### Procedure

The eligibility criteria for people to participate in the study was that they had to: (1). Self-report that they received a diagnosis from a health professional for vaginismus based on criteria in the ICD-10, which was in usage during recruitment; (2) Live in Australia; and (3) Be aged 18 years or above. There was no exclusion around gender identity to allow gender diverse people who met all other criteria for inclusivity following a feminist approach. The recruitment information included a summary of the study, eligibility criteria, $25 voucher incentive, and contact email.

This recruitment information was displayed on bulletin boards and in public women’s bathrooms at Western Sydney University. The first author contacted three national diversity organisations, four national sexual health organisations, and four physiotherapy clinics in Sydney and Perth to help with recruitment. The first three authors also circulated the study information on their personal and professional social media accounts on Facebook, Twitter, and LinkedIn. The first author posted the study information to one Facebook vaginismus support group and eight support groups focused on vaginismus or sexual health on Reddit. Fifty-four people contacted the first author via email to indicate their interest to participate in the study. They were then emailed the participant information sheet and consent form before agreeing to participate. Twenty-one people responded and consented to be interviewed which formed the sample of this study.

### Data collection

The semi-structured interview guide was developed for the larger study which was informed by feminist theory. The flexibility of semi-structured interviews suited a feminist focus to empower participants to voice their perspectives and experiences in their own words (Hesse-Biber, 2007; Liamputtong, 2020). A five-step process by Kallio, Pietilä (Kallio et al., 2016) was followed to develop the semi-structured interview guide with rigour. This involved the first author undertaking a systematic literature review to retrieve knowledge to develop the preliminary interview guide questions (Kallio et al., 2016). After undergoing internal testing with co-authors, the interview guide was pilot and field tested with the first five participants (Kallio et al., 2016). This led to additional questions being added to subsequent interviews and emailed to the first five participants for consistency, and their emailed responses were pasted to their transcripts. The full interview guide with demographic questions is presented in Supplementary Table 1 (Kallio et al., 2016).

From January–May 2020, the first author, who had postgraduate training in qualitative data collection methods, conducted and audio-recorded interviews using Zoom videoconferencing software to circumvent geographical distances and travel expenses. As part of the interview situation, it was verbally confirmed that participants had adequate privacy before beginning the interview. The eligibility criteria were adapted to interview one overseas participant on only her previous help-seeking experiences when she lived in Australia. The interviews ranged from 54 min–2 h and 54 min. To uphold data safety, the first author used her institutional Zoom account to create meeting links. The first author sent the links to participants with her institutional email account as per the terms of the ethics approval. Participants were provided password protected links to access Zoom for the interview to ensure security as no one without the password could join.

Trint transcription software was used to transcribe the Zoom audio-recordings of interviews verbatim. The first author then manually reviewed and verified transcripts against the audio files. As part of member-checking, participants were emailed their transcripts to review for accuracy before data extraction began (Sahakyan, 2023; Varpio et al., 2017). A naturalised approach to transcription was undertaken to accurately capture participants’ expression with its idiosyncrasies of speech such as pauses or word repetition (Nascimento & Steinbruch, 2019). This was done to avoid altering a marginalised group’s expression in line with a feminist approach (Davidson, 2009). Pseudonyms were used for all participants to ensure their privacy.

### Sample

The sample of participants consisted of 20 women and one person who identified as agender. Their age ranged from 19 to 37 years old with a mean of 27.6 years. While 17 participants identified as heterosexual, four others identified as non-heterosexual. Fifteen participants came from White/Anglo/Caucasian backgrounds while the remaining six participants were ethnically and culturally diverse. Most participants sought help in metropolitan areas while three undertook help-seeking for vaginismus in rural areas. The demographic details of the sample are presented in Table 1.

**Table 1.** Demographic information of participants.

Demographic category	***n***	Demographic category	***n***
**Gender**		**Ethnicity**	
Woman	20	White, Caucasian, Anglo-Saxon/Australian, or European	15
Agender	1	Filipino	1
**Sexual orientation**		African	1
Heterosexual	17	Middle Eastern	1
Bisexual	2	Dutch	1
Pansexual	1	Indian	1
Greysexual	1	Vietnamese	1
**Religion**		**Vaginismus type**	
No religion	7	Primary	14
Anglican Christian	1	Secondary	3
Catholic	3	Primary and secondary (recurrent)	1
Lapsed Catholic	1	Unclear	3
Buddhist	1	**State where help was sought**	
Muslim	2	NSW	9
Atheist	3	SA	2
Agnostic	1	WA	1
Pagan witch	1	QLD	3
Other or missing	1	VIC	8
**Relationship status**		**Region type**	
Single	8	Metropolitan	21
In a relationship/married	13	Rural	3
**Pelvic pain co-morbidity**		**Highest level of education**	
Endometriosis	8	Highschool	5
Vulvodynia	4	Post-high school qualification – Technical and Further Education (Tafe)	1
Adenomyosis	1	Bachelors/Honours degree	10
Interstitial cystitis	1	Graduate certificate	1
Undiagnosed pelvic pain	1	Masters degree	3
Chronic pelvic pain	2	Doctorate degree	1
Overactive bladder/pelvic floor	1	**Self-stated socioeconomic status**	
**Age**		Upper middle class	6
18–24 years	6	Middle class	14
25–34 years	13	Poor	1
35–44 years	2		
	***Mean***		***Range***
**Age in years**	27.6		19–37

### Data analysis

An abductive approach to thematic analysis was employed as a feminist tool to categorise participants’ experiences of dis/empowerment (Jenkins et al., 2019; Clarke & Braun, 2019). To ‘revisit the phenomena’, the first author listened to the audio recordings of the interviews and transcripts were each read three times (Timmermans & Tavory, 2012). This allowed the first author to be immersed in the data and illuminated previously unrealised facets from conducting the interviews (Timmermans & Tavory, 2012). For ‘defamiliarisation’, the first author’s repeated review of transcripts as textual records of the atextual interviews created distance and estrangement from it (Timmermans & Tavory, 2012). Such defamiliarisation crystallised aspects that were skimmed over during the live interviews (Timmermans & Tavory, 2012). Through revisiting the data and defamiliarising from it, the first author developed preliminary parent codes (Timmermans & Tavory, 2012).

As part of ‘alternate casing’, all authors applied multiple theories to make sense of cases of data (Timmermans & Tavory, 2012). Gender identity theory and theories of heteronormativity were considered to rethink data. However, it was the larger study’s feminist frame which highlighted women’s constructions of self in relation to dis/empowerment towards womanhood, agency, and resilience when help-seeking. This informed revision of parent codes for themes. While initially theories of self-esteem and self-efficacy were applied to explain the association with women’s help-seeking for vaginismus, it was too limiting. Therefore, theory of self was used to illuminate the broad and nuanced healthcare implications of help-seeking and sense of self when developing child codes (Gallagher, 2013). A constructivist philosophy underpins the thematic analysis (Naeem et al., 2023). It informed the labelling of codes to categorise participants’ subjective interpretations (Naeem et al., 2023). Authors were thus able to develop code names that captured participants experiences of self in relation to their dis/empowerment, self-critique of their constructions, and its implications on subsequent health behaviours.

The first author then used Quirkos software to code every line of the transcripts from the alternate casing. All authors then reviewed the Quirkos coding to merge, delete, or add new codes (Rodik & Primorac, 2015; Spangler, 2019). Sample size adequacy was reached at 18 participants (Vasileiou et al., 2018). No new information on participants’ help-seeking for vaginismus and healthcare implications on sense of self emerged in the subsequent interviews (Vasileiou et al., 2018). This aligns with research which finds that 9–17 interviews with participants provide a sufficient qualitative sample size (Hennink & Kaiser, 2022). Data from the demographic questions was analysed using descriptive statistics.

### Qualitative rigour

For the accurate recording of phenomena for credibility, each interview transcript was spot-checked by the first author and member-checked by participants (Rowlands, 2021; McMullin, 2023). To achieve transferability and dependability to assess findings and replicate the study, the interview guide, data collection strategies through recruitment, data analysis, and theory have been detailed (Adler, 2022). For the confirmability of findings, internal and field testing of the interview guide reduced the authors’ subjectivity in its development (Kallio et al., 2016). The number of participants’ statements that informed each subtheme and participants’ quotes are presented in the results to demonstrate that the analysis is data driven as part of confirmability (Nowell et al., 2017).

### Researcher reflexivity

Abductive reasoning rests on researchers’ repertoire of theory to inform reflexivity (Timmermans & Tavory, 2012). The second, third and last authors’ health professional backgrounds in psychology, physiotherapy, sex therapy/medicine respectively shaped the healthcare-focused analysis and consideration of clinical co- and multi-morbidities. Conversely, all the authors’ personal experiences help-seeking for sexual and reproductive health issues as patients illuminated the androcentrism of the healthcare system and the insight to be gained from a feminist approach. The first two authors’ proficiency in heteronormative concepts and theories of self has informed the gendered analysis of womanhood and femininity. All authors are ethnically and culturally diverse with Indian, Zimbabwean and Western European heritage with varying religious upbringings and beliefs. This allowed the researchers to use a feminist lens to recognise how culture and religion dis/empowered the ethnically, culturally and religiously diverse participants’ help-seeking and its health implications on their sense of self. Therefore, the mixed researcher positionalities helped to balance analysis and inform critical discussion for rigour.

## Results

Three main themes were developed: *Interconnected constructions of womanhood and help-seeking*, *Elicit agency to move forward*, and *Resilience to surmount challenges*.

### Interconnected constructions of womanhood and help-seeking

Participants’ help-seeking and constructions of womanhood informed their subsequent health behaviours. It led to their *Self-critique of being a real woman* and to *Negotiate femininity and sexual well-being*. This affected their future help-seeking approaches.

#### Self-critique of being a real woman

Nineteen participants’ self-critiqued their perceptions of being a real woman. When asked how help-seeking and undergoing treatment for vaginismus impacted her construction of self, Chloe responded that ‘It was really, really difficult. I felt like less of a woman, that I wasn’t a real woman.’ Not feeling like a real woman informed participants’ explicit self-critique of being a ‘broken’ woman who needed to be ‘fixed’ with treatment to achieve painless penis-in-vagina (PIV) sex. After enduring long periods of treatment without anticipated progress, Clara shared that:

… you feel [2 s pause] like you’re a broken body … it kind of takes away your motivation as well. So I think it just like makes you to reposition your mindset to like valuing getting better a little bit less.

Clara expressed nine other women’s self-critique that feeling broken and not experiencing treatment benefits reduced their self-motivation and devalued help-seeking.

Fourteen participants divulged that mental health support, empathetic care from treating health professionals (HPs), and seeing other successful and attractive women also help-seeking for vaginismus in online support groups reframed their constructions of womanhood. Participants noted that they found that other women, who despite upholding feminine ideals of attractiveness and success, struggled with vaginismus.

This enabled participants such as Crystal to realise that ‘that there is no such thing as a true female’. They then critically evaluated their constructions of womanhood to understand that there were no unchanging set ideals of femininity and womanhood. This allowed them to positively self-critique and accept themselves as women even when they had vaginismus. Even five women who did not anticipate being pain-free, due to their pelvic pain co-morbidities, believed that they were not lacking femininity without PIV sex. They productively self-critiqued and accepted themselves as normal women, which they said enabled them to continue help-seeking and treatment.

#### Negotiate femininity and sexual well-being

Out of guilt, six women compromised their own sexual well-being to endure pain to have PIV sex with their partners. They noted it worsened their symptoms of vaginismus. Since most participants associated sex with pain, ten of them tended to refrain from sex and intimacy. Jasmine explained the impacts of refraining from sex due to vaginismus:

You don’t want to do anything that makes you feel in pain so massively. And that’s had like an impact on my self-esteem as well. Because you just don’t feel desirable. You don’t feel like a sexual being at all, you just feel like a human, not a woman.

As Jasmine illuminated, women protected their self from pain by refraining from sex. However, this compromised their femininity by making them not feel like a sexual ‘woman’ but as a desexualised ‘human’ as Jasmine noted.

Through HPs’ assistance, six women negotiated their understandings of sex simply being PIV. Molly shared how consulting a sex therapist was helpful:

for me to be able to work through my perception of what sex is and what it includes and then sort of to come to a level of acceptance of, OK, this is what it is … And, you know, I might not be able to do this particular activity [PIV sex], but I can still be intimate with my partner in all of these other ways.

Through appropriate healthcare, participants like Molly negotiated their perception of PIV sex and gained satisfaction without it weakening their femininity. Molly had additional chronic pelvic pain. Consequently, her HPs noted that fully painless PIV sex may not be possible. She continued that one should seek ‘other things that can still give you the same intimacy or satisfaction or whatever it might be. And just manage it rather than trying to fight it.’ This negotiation of femininity and sexual well-being drove several women to continue following help-seeking to manage the condition, even if cure was not possible.

### Elicit agency to move forward

Women who elicited their self-agency to move forward could *Traverse* the *mental and emotional rollercoaster* of help-seeking for vaginismus. Their *Perception of control to overcome vaginismus* informed their subsequent help-seeking approaches. Participants’ *Cultural and religious background influence seeking support* for their symptoms.

#### Traverse mental and emotional rollercoaster

Participants shared that experiencing pain is draining, they did not receive the answers they hoped for when help-seeking, and/or felt unheard by HPs. This made traversing the help-seeking process ‘stressful,’ ‘upsetting’, a ‘rollercoaster’ and/or ‘depressing’ according to 19 participants. It destabilised their sense of self-agency and exacerbated some women’s pre-existing mental health issues or caused it. Grace reflected: ‘I feel like health professionals often don’t realise that things that they say and do, even like one appointment, can have such a big impact on how the patients view themselves after a long time.’ As Grace highlighted, the offhand comments that HPs said affected many women’s mental and emotional well-being in ways that shaped their sense of self and approaches to future help-seeking. For example, Rita shared how a HP stated that men who attempted to have sex with her would feel like they ‘were hitting a brick wall.’ She referred to how this offhand comment ‘psychologically impacted’ her and shaped her embarrassed and sexually avoidant self to avoid discussing her condition or seeking sexual encounters.

Nonetheless, according to 19 participants, HPs also helped them to traverse the mental and emotional rollercoaster of help-seeking in ways that strengthened their self-agency to follow treatment. Olivia explained why she felt motivated to follow treatment:

I didn’t want to let her [HP] down, I know that’s stupid, it’s my own health. But like, I felt like she believed in me enough that I could do it. So that gave me more promise to do it because I didn’t want to let her down. Even though it was like, if I didn’t do it, I was letting me down.

In line with other women’s accounts, Olivia’s comments illuminated how a HP who believed in her ability to overcome vaginismus empowered Olivia to believe in herself. Participants shared how this drove their self-efficacy to not give up on treatment and traverse the rollercoaster nature of help-seeking.

#### Perception of control to overcome vaginismus

All participants’ perception of control to overcome vaginismus varied depending on their HP’s approach to help and/or their treatment progress. While eight participants stated that they felt they had control to overcome vaginismus, they varied their answer when asked if there were any other factors that impacted their perception of control. They then referred to how their HPs’ level of willingness to support them influenced their perception of control regarding help-seeking to overcome vaginismus. For instance, Hannah shared that:

I think I didn’t really feel in control because of the fact she [GP] said I was kind of just the way I was, that I kind of had to deal with it. So that’s not really feeling like being in control but more like that there’s nothing you can do about it.

Before gaining her diagnosis of vaginismus, a GP incorrectly told Hannah that her vaginal canal was too narrow, and nothing could be done. HPs’ discouraging advice that help-seeking is futile led to Hannah and nine other women’s perception of having no control over the situation. As Hannah stated, such discouraging HP advice reduced these women’s sense of self. This led to them taking a break from, or ceasing help-seeking, due to believing that they had no control to overcome vaginismus.

When making treatment progress, fourteen women felt as though they were reclaiming the lost control over their bodies and pelvic floor muscles. According to Clara, receiving tangible exercises and strategies to treat vaginismus meant that ‘You have control over your future now, like you can do something about it now. You don’t have to be like a victim or patient anymore.’ Similar to Clara, participants shared how prior to receiving help and treatment progress, their perceived loss of control over their bodies weakened their sense of self to feel like a ‘victim’. However, seeking and receiving help empowered them to perceive control over their situation to overcome vaginismus and improve their future.

#### Cultural and religious background influence seeking support

Five participants self-identified as not being of an Anglo/Caucasian/European/White background. One participant shared that her religious garb and background led to covert stereotyping from her HP. However, all five participants revealed how their diverse backgrounds shaped their help-seeking. Participants who self-identified as White, Caucasian, European, or Anglo also noted how their religious upbringing informed their help-seeking and sense of self.

Religious and cultural values impacted participants’ perceptions about discussing sexual problems, which informed their help-seeking approaches. Eleven participants referred to growing up in religious or cultural households where sex or sexual problems were not discussed. Anna was one such participant who shared that:

… my mum grew up in the Philippines and they’re a very Catholic country. So her upbringing, you know, periods or painful sex or sex in general wasn’t something that they spoke about openly. So, in a way it has affected me because she has been like that towards me growing up … I think that’s why perhaps the diagnosis, for example, went on for so long because I wasn’t brought up in an environment where it was encouraged to speak about.

Anna’s experience was shared by other women who noted that the influence of the Catholic environment that they grew up in, whether it be from parents and/or schooling, promoted the silencing of discussing sexual matters. However, after they began to action help-seeking, some women became self-aware about their reserved attitudes toward sex similar to Anna’s statement. They then negotiated it to seek help more confidently and comfortably.

In contrast, three women’s religious views aided their help-seeking journeys and strengthened their sense of self. Chloe who identified as a pagan witch shared how:

A large part of paganism is questioning the status quo; not just accepting tradition and authority, but deconstructing and deciding for oneself. This type of worldview helped me overcome the negativity and unhelpfulness of certain medical practitioners. I didn’t just accept what they said, I questioned it and decided for myself. I have also used various pagan practices to help me in my journey – meditation whilst dilating, spells/rituals for motivation and so forth.

Like Chloe, these participants credited their religious views to help construct self-agency to not simply accept certain HPs’ negative advice but question it to decide the right path forward when help-seeking.

### Resilience to surmount challenges

Participants who evoked resilience were able to surmount challenges. To do this, they had to elicit the *Perseverance to improve* their *situation* and *Comparative coping to maintain positivity*. This fostered their self-resilience to grapple with long help-seeking.

#### Perseverance to improve situation

Fifteen women explained their motivations to persevere with help-seeking even when faced with barriers to treatment and health professional practice. When asked what made her persevere and seek help when a HP did not believe her vaginismus diagnosis, Amy responded:

… realising that I was worth more than that, and that I deserved to be treated with respect. And also just even feeling the worsening of symptoms and going actually ‘No, I don’t think this is all in my head. This is something that I’m still dealing with.’ That was really important for me to realise. And I think it was a nudge. It was like ‘okay this is still here. This isn’t going away.’ Let’s do something about it.

Once women such as Amy perceived their self-worth, it drove their perseverance for self-improvement to ‘do something about it’. This included searching the internet for reviews on HPs, browsing recommendations in support groups, and reaching out to other healthcare facilities to continue help-seeking for vaginismus when not satisfied with past medical advice.

For eleven women, their relationship status of being in a relationship or single informed their self-perseverance to either resume or halt help-seeking respectively. After facing challenges and stopping treatment, six women admitted that their self-perseverance to restart their help-seeking journeys was to alleviate their guilt towards their partners and improve intimacy. Conversely, five women such as Belle stated: ‘I’m single now. So I guess I like, I don’t really need to dilate because I have no need for it.’ Their demotivated self did not view help-seeking as a priority to persevere with treatment due to being single.

#### Comparative coping to maintain positivity

Eleven women practiced comparative coping. They compared their challenges of vaginismus to what they perceived as worse circumstances to cope with the taxing nature of help-seeking. They rationalised that everyone has problems, which could be worse than help-seeking for vaginismus. Belle expressed such a rationale when she explained:

… no one will ever make jokes about me being the old lady with a loose vagina. So I think I kind of look at it like in that positive sense. I mean, it could be so much worse. Like, I could be legitimately like really sick and have like something wrong with me.

Participants like Belle practiced comparative coping to strengthen their self-resilience. This helped them to recognise the magnitude of their condition while putting it in perspective to positively accept and endure the long haul of help-seeking.

When faced with treatment stagnations or regression, seven participants compared themselves to others who made less progress than them which they heard from HPs or read in online support groups. Kate articulated this when she explained: ‘I knew other women who were like talking about "oh, I finally was able to use a cotton bud today." And I'm like, that's not me. Like I can insert the third dilator. That was motivating.’ Such comparative coping facilitated women’s constructions of their self-achievement with treatment progress to feel motivated enough to continue help-seeking despite facing roadblocks.

## Discussion

**Figure 1.**
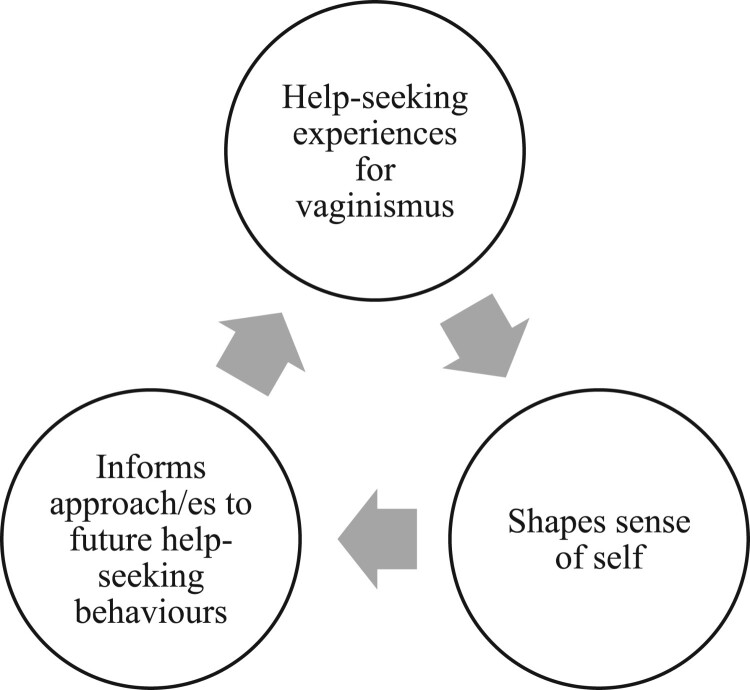
Interrelationship between women’s help-seeking for vaginismus and their sense of self.

Timmerman and Tavory’s formal methodological steps for qualitative abductive reasoning were followed to investigate the surprising missing research on the relationship between help-seeking for vaginismus and healthcare implications to their self (Timmermans & Tavory, 2012). By revisiting the phenomena, defamiliarising from it, and applying alternate casing, a feminist approach to theory of self led to the following novel explanation (Timmermans & Tavory, 2012). The nature of participants’ help-seeking experiences drove their constructions of a dis/empowered self. This informed their approach to subsequent help-seeking and vice versa like a feedback loop illustrated in the model in Figure 1. The approach to subsequent help-seeking behaviours includes participants’ response to gain diagnosis, follow treatment, gain information, and seek support. While scant studies have examined help-seeking for vaginismus (Ogden & Ward, 1995; Bond et al., 2012), a few other studies have considered the effect of vaginismus or dyspareunia on self-esteem or self-worth (Ward & Ogden, 1994; Stelko, 2015; Facchin et al., 2021; Engman et al., 2010). However, no study has examined both aspects of the interrelationship between people’s help-seeking for vaginismus and sense of self, and its implications on their future health behaviours. Therefore, this study’s finding presented in the model of Figure 1 is not fully accounted for by existing theories, which demonstrates an original abductive contribution (Timmermans & Tavory, 2012).

There is a lack of research that directly reports on how women’s help-seeking for vaginismus influenced their sense of self (Pithavadian, Chalmers et al., 2023a). Ward and Ogden (Ward & Ogden, 1994) explored women’s self-perception when having vaginismus and Engman, Wijma and Wijma (Engman et al., 2010) briefly considered the association between cognitive behavioural therapy completion and increased self-worth in women with vaginismus. However, no prior study examined how participants’ *Interconnected constructions of womanhood and help-seeking* for vaginismus shaped their disempowered sense of self. Theory of self, particularly self-discrepancy, has not been applied to help-seeking for vaginismus (Pithavadian, Chalmers et al., 2023a). The results indicate that there is a discrepancy between participants’ ideal self and perceived self (Watson & Watts, 2001). Such discrepancy weakens women’s sense of self, and they feel disempowered and unsure of themselves as women and sexual beings as reported in the results (Flury & Ickes, 2007). This reduces women’s belief in their own power and capacities (Flury & Ickes, 2007). It can shape their subsequent approach to doubt whether help-seeking will ever enable them to attain their ideal self. This could cause them to view treatment as pointless and halt it (Higgins, 1987). It can potentially reinforce their disempowered sense of self and perpetuate their reticent approach to help-seeking and vice versa like a feedback loop.

Research has found that women with vaginismus and other dyspareunic co- and multi-morbidities feel that they lack femininity or womanhood (Facchin et al., 2021; Ayling & Ussher, 2008; Shallcross et al., 2018; Marriott & Thompson, 2008). Diverging from that, several participants in this study negotiated their ideals of womanhood and realised that there was no ‘real’ woman and broadened perceptions of sex beyond PIV. This reduced the gap, or self-discrepancy, between their ideal self and perceived self in relation to womanhood (Higgins, 1987). It empowered women to reconstruct a stronger sense of self to be certain of their abilities to gain motivation and successfully take action to move forward and surmount challenges during treatment (Watson & Watts, 2001; Bak, 2014).

Upon being unable to enact the idealised heteronormative PIV sex script, participants had constructed a disempowered self (Sörensdotter, 2017). This aligns with Fahs (2011) work which contends that women’s bodies behold power dynamics that privilege men’s sexual desires. In fact, studies indicate it is common for women to endure painful sex for men’s sexual needs (Fahs, 2011; Carter et al., 2019; Elmerstig et al., 2008). Fahs (2011) notes that heterosexual women find PIV sex to be the most satisfactory even when it does not give them sexual pleasure. Heterosexual women instead gain pleasure from their male partners’ orgasm through enacting the PIV sex script (Fahs, 2011). This reinforces the status quo of women’s pleasure being linked to their sexual powerlessness to endure painful sex for their male partner (Fahs, 2011).

Fahs (2011) argues that women can also gain pleasure and empowerment by rejection of the heteronormative PIV-centred script. In line with this, some participants deprioritised PIV sex and reframed their perceptions of sex being more than PIV. It strengthened their sense of self as women and sexual beings. This has not been reported in the literature specific to vaginismus or SPPD (Pithavadian, Chalmers et al., 2023a). However, even with related dyspareunic conditions, Shallcross et al.’s (2018) review found only one woman with vulvodynia who adopted an ‘egalitarian relational discourse’ (p. 591)to work with her partner to experiment and enjoy non-PIV sexual practices. Therefore, reframing perceptions of PIV sex could be promising to empower women with vaginismus or SPPD (Fahs, 2011), and improve their self and motivation for future help-seeking. However, it should be noted that Shallcross et al.’s (2018) one participant and this study’s participants who exercised such reframing to reclaim their lost power shared a commonality. They either had a male partner, or began or resumed help-seeking after a break to improve sexual relations with men (Bak, 2014). This indicates that rather than being a solely internal process within heterosexual women, they still find male partners’ support and involvement to be integral for them to successfully reframe their heteronormative scripts of PIV sex.

Other factors like religion and health professionals also influenced help-seeking. While research on dyspareunic conditions tends to focus on how religious restrictions can hinder help-seeking (Azim et al., 2021), this study uniquely considers how religion, such as paganism, empowered some participants and improved their sense of self to persevere through the challenges of help-seeking. Moreover, mental health support and empathetic care from HPs enabled women to close the gap between their ideal and perceived selves. This improved their self-worth to endure their trying help-seeking journeys (Watson & Watts, 2001).

However, such positive outcomes from HPs are not always the case as studies have indicated that some HPs offer dismissive or invalidating responses that did not believe women’s experiences of painful sex (Hildenbrand et al., 2021; Hintz, 2023; Braksmajer, 2018). Alternatively, this study found that even when some HPs believed women’s diagnosis of vaginismus, they were discouraging, even in offhand comments, to women’s prognosis and treatment. This can dis/empower women’s sense of self for readiness to change their health by help-seeking (Skuladottir & Halldorsdottir, 2008; Holt et al., 2010). It shaped certain participants’ disempowered self that regressed and stopped taking action and maintaining the change of help-seeking (Freeman & Dolan, 2002; Person & Finch, 2009). This causes a feedback loop of one’s discouraging help-seeking experiences leading to a disempowered self, which shapes the subsequent help-seeking approach to quit. In some cases, HPs’ discouragement may be unintentional and the result of compassion fatigue, being overworked, and not receiving enough support from their institutions of employment (Knaak et al., 2017).

However, HPs’ discouraging comments can also be informed by the patriarchal attitudes of the healthcare system that tend to doubt women’s concerns, knowledge of their own bodies, and their power and strength to overcome challenges (Shallcross et al., 2019). This leads to women’s complaints of painful sex or their efforts and resolve to improve their health to be silenced and negated (Shallcross et al., 2019). Consequently, people with vaginismus are deprived of their sexual and reproductive health and right (SRHR) to access and receive appropriate medical treatment and support without covert patriarchal discrimination (Pithavadian, Mpofu et al., 2023c). Lacking or compromised SRHR for people with vaginismus reduces their agency and power, which increases negative help-seeking experiences (Pithavadian, Mpofu et al., 2023c). This can diminish their sense of self and level of motivation for future help-seeking like a feedback loop.

### Implications for practice and research

The study’s finding of the interrelationship between help-seeking for vaginismus, sense of self, and subsequent health behaviours has implications for practice and research. HPs can be trained in the skills to offer support, through statements, body language and building trust, to help those with vaginismus to overcome the gap between their ideal and perceived selves (Gabay, 2019). This empowers women’s self to persevere with help-seeking without feeling lesser to experience improvement. Women’s comparative coping, especially on online social mediums, highlights how it can be used as another avenue, besides HPs, to help women renegotiate their self-ideals (Jiang & Ngien, 2020). It can empower their self-worth to continue help-seeking to achieve relief even when they face difficulties.

HPs should work on rewriting heteronormative sex scripts that exalt PIV sex with their patients who have vaginismus (Farrell & Cacchioni, 2012; Cacchioni, 2007). HPs can do this to give people with vaginismus the confidence and power to not force themselves to endure pain and have PIV sex for the satisfaction of their sexual partner. It can especially be helpful during long waiting periods to see HPs or access treatment such as Botox injections that can deter women from help-seeking (Alrasheedi et al., 2019). Moreover, rewriting heteronormative scripts can help women with pelvic pain co-morbidities, which has complicated their vaginismus and reduced the possibility of achieving PIV sex.

HPs should focus on women’s holistic health when treating vaginismus and respectfully enquire about patients’ background and its impact on their sexual history (Azim et al., 2021; Donaldson & Meana, 2011). It can aid HPs to identify and address any sociocultural barriers from patients’ background to maintain their momentum or strengthen their sense of self to action help-seeking (Holt et al., 2010). Further study is needed on how certain aspects of religion may positively shape women’s help-seeking and sense of self to better support more religious patients. Future research should seek to uncover and address the barriers that both patients and HPs experience when treating vaginismus. This can support HPs to provide their patients with positive help-seeking experiences for vaginismus to empower their self in ways to maintain their readiness to change until it is achieved.

### Limitations

The association between women’s help-seeking, sense of self, and implications on future help-seeking behaviours should be researched with larger samples of participants. Eligible individuals without stable internet access would have been unable to participate in the study. The interviews being conducted on Zoom videoconferencing software may have been discouraging to talk freely for those who were not familiar or comfortable with using online platforms. For inclusivity, the words ‘participants/people/patients’ instead of gendered pronouns were used to refer to findings that included the one participant who identified as agender. While the authors recognise that any person with a vagina can develop SPPD or vaginismus irrespective of their gender identity, almost all participants’ identification as cis-gendered women means that the study’s findings do not represent gender diversity. Most participants identified as heterosexual and therefore not much sexual diversity informed the study’s findings.

## Conclusion

The existing literature on vaginismus tends to be clinically focused on the affected genitalia. This is the first study to examine how women’s help-seeking for their vaginismus impacts their sense of self. The study contributes novel feminist perspectives to theory of self to uncover the ways that women negotiated dis/empowerment when help-seeking for their vaginismus, and its implications on their future help-seeking and treatment. Participants’ help-seeking experiences impacted their sense of self in ways that informed their approach to future help-seeking, potentially like a feedback loop. Negative help-seeking experiences with health professionals, to gain diagnosis, treatment or support weakened participants’ sense of self. This led to their demotivation or withdrawal from help-seeking. Conversely, positive help-seeking experiences strengthened participants’ sense of self to believe in their own capacities to persevere with treatment even when it was challenging. Women’s weakened sense of self was often caused by a gap between their ideal and perceived self, which led to negative feelings and responses to subsequent help-seeking. Health professionals should be trained to identify possible gaps between ideal and perceived selves in people with vaginismus to offer appropriate support to help them overcome the gap. This can help women to empower their sense of self to accept their situation, own worth, and motivate them to continue help-seeking to experience improvement.

## Ethics approval and consent to participate

This study was approved by the Human Research Ethics Committee at Western Sydney University (Approval Number: H13618). All participants gave informed consent to participate in the study.

## Consent for publication

All participants gave informed consent for publication.

## Availability of data and materials

The dataset generated and/or analysed during the current study are not publicly available as per the conditions of the ethics approval. Information about the data is available from the corresponding author upon reasonable request. Data is located in restricted access data storage at https://doi.org/10.26183/sw56-z237.

## Competing interests

The authors declare that they have no competing interests.

## Supplementary Material

Supplementary Table.docx
